# Meta-analysis of ^18^ F-PSMA-1007 PET/CT, ^18^ F-FDG PET/CT, and ^68^Ga-PSMA PET/CT in diagnostic efficacy of prostate Cancer

**DOI:** 10.1186/s40644-023-00599-y

**Published:** 2023-08-21

**Authors:** Wenxiao Yu, Ming Zhao, Yingjun Deng, Shengjing Liu, Guanchao Du, Bin Yan, Ziwei Zhao, Ning Sun, Jun Guo

**Affiliations:** 1 Department of Andrology, Xiyuan Hospital of China Academy of Chinese Medical Sciences, No.1, R. Xiyuangcaochang, District Haidian, Beijing, 100091 China; 2 Post-doctoral Research Station, Xiyuan Hospital of China Academy of Chinese Medical Sciences, No.1, R. Xiyuangcaochang, District Haidian, Beijing, 100091 China; 3 Graduate School, Beijing University of Chinese Medicine, 11 North Third Ring East Road, Chaoyang, Beijing, China

**Keywords:** Prostate cancer, Prostate-specific membrane antigen, Diagnosis, PET/CT, Radioisotopes, meta-analysis

## Abstract

**Objective:**

To compare ^18^ F-PSMA-1007 PET/CT, ^18^ F-FDG PET/CT and ^68^Ga-PSMA PET/CT in the diagnostic value of prostate cancer.

**Method:**

The Chinese and foreign databases, such as Pubmed, Cochrane Library, Embase, CNKI, VIP, Wanfang, etc., were systematically searched within the period from the establishment of the database to June 1, 2022. Clinical studies related to the diagnosis of prostate cancer by methods such as ^18^ F-PSMA-1007 PET/CT, ^18^ F-FDG PET/CTCT, ^68^Ga-PSMA PET/CT, were researched. Two (2) investigators independently screened literatures, extracted data, and assessed the risk of bias when these data were included in the studies with the Quality Assessment of Diagnostic Accuracy Studies (QUADAS-2). Review Manager5.4, Stata 14.0, and Meta-disc 1.4 software were used for meta-analysis to compare the efficacy of different methods in the diagnose of prostate cancer.

**Results:**

Twenty-seven (27) studies, including 2891 subjects were included in our study. Meta-analysis results showed that the pooled sensitivities of ^18^ F-PSMA-1007 PET/CT, ^18^ F-FDG PET/CT, and ^68^Ga-PSMA PET/CT were 0.912 (95%CI: 0.883–0.936), 0.748 (95%CI: 0.698–0.795), and 0.916 (95%CI: 0.896–0.934), respectively; the pooled specification were 0.878 (0.844–0.907), 0.639 (95%CI: 0.589–0.687), and 0.734 (95%CI: 0.685–0.779), respectively; the positive likelihood ratios were 6.335 (95%CI: 4.288–9.357), 2.282 (95%CI: 1.497–3.477), and 3.593 (95%CI: 2.986–4.323), respectively; the negative likelihood ratios were 0.878 (95%CI: 0.844–0.907), 0.374 (95%CI: 0.280–0.499), and 0.110 (95%CI: 0.083–0.144), respectively; the diagnostic odds ratios were 65.125 (95%CI: 34.059–124.53), 7.094 (95%CI: 4.091–12.301), and 29.722 (95%CI: 20.141–43.863), respectively; the positive posterior probability was 64%, 38%, and 62%, respectively; the area under the SPOC curve was 0.95 (95%CI: 0.93–0.97), 0.81 (95%CI: 0.78–0.84), and 0.96 (95%CI: 0.92–0.98), respectively. The funnel plots indicated that there was no significant publication bias in the included literatures.

**Conclusion:**

The current evidences showed that ^18^ F-PSMA-1007 PET/CT and ^68^Ga-PSMA PET/CT had higher diagnostic efficacy of prostate cancer compared with ^18^ F-FDG PET/CT, among which ^68^Ga-PSMA PET/CT was slightly higher in the sensitivity of the diagnosis of prostate cancer, while ^18^ F-PSMA-1007 PET/CT may have higher efficacy in specificity and confirmed positive rate. Due to the limitations of the quality of the included samples and literatures, the above conclusions should be further validated by expanding the sample size and improving the quality.

## Introduction

Prostate cancer (PCa) is a common genitourinary malignant tumor and the fifth leading cause of death in men due to cancer [1]. The survey in 2018 showed that there were about 1.3 million new cases worldwide and about 359,000 related deaths about PCa [2]. In recent years, the incidence of prostate cancer has been increasing with the aging of the population, and the challenges in the related health resources are also becoming more and more severe. The prostate cancer has an insidious onset in the early stage, and is lack of specificity in clinical manifestations. Most patients are often accompanied by invasion and metastasis when they have clinical symptoms. As a common malignant tumor leading to the death of men worldwide, the pathological characteristics and clinical manifestations of prostate cancer often have significant heterogeneity, which is reflected in not only different individuals, but even the same patient [3, 4]. Although the diagnosis and treatment of PCa has developed rapidly in recent decades, the highly heterogeneous pathological characteristics of PCa increase the difficulty in clinical diagnosis and staging, and are still important factors affecting the early screening of high-risk PCa populations. Medical imaging examinations have always played an important role in the diagnosis and treatment of PCa. As the treatment protocol for PCa has gradually become more individualized in recent years, the selection of imaging methods is critical to accurately assessing the diagnosis, staging, and retesting of PCa patients.

Clinically, the diagnosis, staging, and bone metastasis of PCa mainly rely on the detection of serum prostate-specific antigen (PSA) test in combination with imaging means such as CT, MRI, and systematic bone scans, which still have the risk of negative or false positive results [5]. In recent years, radionuclide-labeled targeted molecular imaging has shown good prospects in the clinical application of PCa, and has become a key point of the studies on disease diagnosis, treatment, biochemistry and recurrence [6, 7]. As a new diagnostic technology widely used in clinical practice, PET/CT can significantly improve the accuracy of clinical disease diagnosis since it incorporates the advantages of anatomy, functional metabolic imaging and molecular imaging, and has become an important means for diagnosing PCa [8]. Correspondingly, the types of PET/CT imaging agents have gradually increased with the development of PET/CT, such as ^18^ F-PSMA, ^18^ F-FDG, ^68^Ga-PSMA, ^11^c-choline, etc.The application of these imaging agents has improved the sensitivity and specificity of PET/CT in diagnosis of PCa, and prolonged the survival of patients [9, 10].

Prostate-specific membrane antigen (PSMA) is an important target for PET/CT diagnosis of PCa patients. PSMA corresponds to PCa grading and staging in the histopathological expression level. It is related to the invasion, metastasis and recurrence of prostate tumors, helps to diagnose tumors in other organs based on the expression in the neovascular endothelium, and promotes the development of many PSMA ligand-related targeted radiopharmaceuticals at the same time [11]. The nuclide ^68^Ga is the first specific imaging agent used to label PSMA because of the characteristics of high positron energy and short half-life. Studies have confirmed that the PET/CT using ^68^Ga-PSMA was satisfactory in sensitivity and specificity for the diagnosis of PCa. The nuclide 18 F has a longer half-life and better pharmacokinetics, resulting in a higher radioactive uptake rate [12]. ^18^ F-FDG, as the earliest imaging agent used in PET/CT, is involved in the body’s glucose metabolism, and differentiates tumor lesions from other tissues by glucose utilization, which can also better reflect tumor progression [13]. At present, there are differences in energy intake and pharmacokinetics of different imaging agents, and different imaging methods have different diagnostic criteria for PCa, resulting in controversial accuracy for PCa by ^18^ F-PSMA-1007 PET/CT, ^18^ F-FDG PET/CT, and ^68^Ga-PSMA PET/CT. Therefore, this study analyzed and compared ^18^ F-PSMA-1007 PET/CT, ^18^ F-FDG PET/CT, and ^68^Ga-PSMA PET/CT in the diagnostic efficacy of PCa in order to provide more reference and evidences for the selection of clinical imaging examination protocols.

## Materials and methods

### Search strategy

**Fig. 1:**
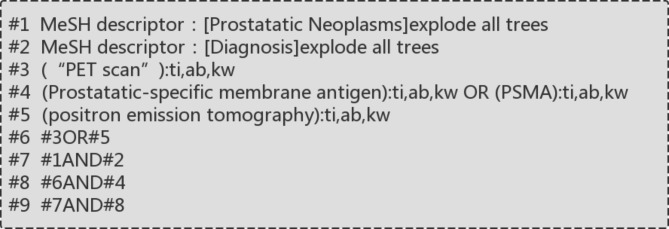
Search strategy for Cochrane Library

The Chinese and foreign databases, such as Pubmed, Cochrane Library, Embase, CNKI, VIP, Wanfang, etc., were systematically searched within the period from the establishment of the database to June 1, 2022, in order to collect data in clinical studies related to the diagnosis of PCa by methods such as ^18^ F-PSMA-1007 PET/CT, ^18^ F-FDG PET/CTCT, ^68^Ga-PSMA PET/CT, etc. A combination of database search and manual search was used to set subject headings/abstract words, including prostate cancer, prostate tumor, prostate-specific membrane antigen, diagnosis, PET/CT, radioisotopes (Chinese, English), etc. The specific search strategy was adjusted according to the characteristics of the database searched. Taking Cochrane Library as an example, the specific search strategy was shown in Fig. 1.

### Inclusion/exclusion criteria

Inclusion criteria: ① Literatures on diagnostic studies of ^18^ F-PSMA-1007PET/CT and/or ^18^ F-FDG PET/CT and/or ^68^Ga-PSMA PET/CT in the detection of primary PCa; ② Before receiving the above imaging examination, the patient did not receive any prostate-related surgery; ③ The pathology test results were used as the gold standards; ④ The paper was written in Chinese or English.

Exclusion criteria: ① Repeated publications; ② Studies without outcome indicators, case reports, overview, conference abstracts, and studies targeted to animals and cells; ③ Literatures from which the data related to the true positive value (TP), the false positive value (FP), the true negative value (TN), and the false negative value (FN) cannot be extracted.

### Literature screening and data extraction

All included literatures were screened independently by two reviewers. Preliminary screening was carried out by reading the article titles and abstracts to exclude irrelevant literatures. According to the inclusion and exclusion criteria established in the study, re-screening was completed after reading the full text, and data were extracted from the literatures, including: first author, publication year, country, sample size, TP, FP, TN, and FN.

### Quality assessment

The QUADAS-2 scale [14] was used as the quality assessment tool to assess the risk of bias and applicability of the literatures. The scale includes four areas including case selection, diagnostic tests to be evaluated, gold standards, and the case flow and the time interval between the diagnostic tests and the implementation of the gold standards. Risks in each area were assessed as Low Risk, High Risk, and Unclear Risk. Two reviewers independently assessed the risk of bias in the included literatures, cross-checked the assessment results, and resolved controversial results by discussion or third-party review.

### Statistical analysis

Statistical analysis was performed using Review Manager5.4, Stata 14.0, and Meta-disc 1.4 software. The literatures related to ^18^ F-PSMA-1007PET/CT and/or ^18^ F-FDG PET/CT and/or ^68^Ga-PSMA PET/CT were calculated for pooled sensitivity (SEN), pooled specificity (SPE), positive likelihood ratio (LR+), negative likelihood ratio (LR-), diagnostic odds ratio (DOR), and positive posterior probability (PPP), respectively, plotted for the Summary Receiver Operating Characteristic (SROC) and calculated for the area under the curve. Q-test and I2 were used to test for heterogeneity. When both p > 0.1 and I^2^ ≤ 40% were satisfied, a fixed effects model was used. A random effects model was used considering heterogeneity among studies. Moreover, Meta regression analysis was used to identify the potential source of heterogeneity. Meta-analysis level α was setted as 0.05; Deek’s funnel plots were drawn to test for publication bias.

## Results

### Literature screening results and general characteristics

**Fig. 2:**
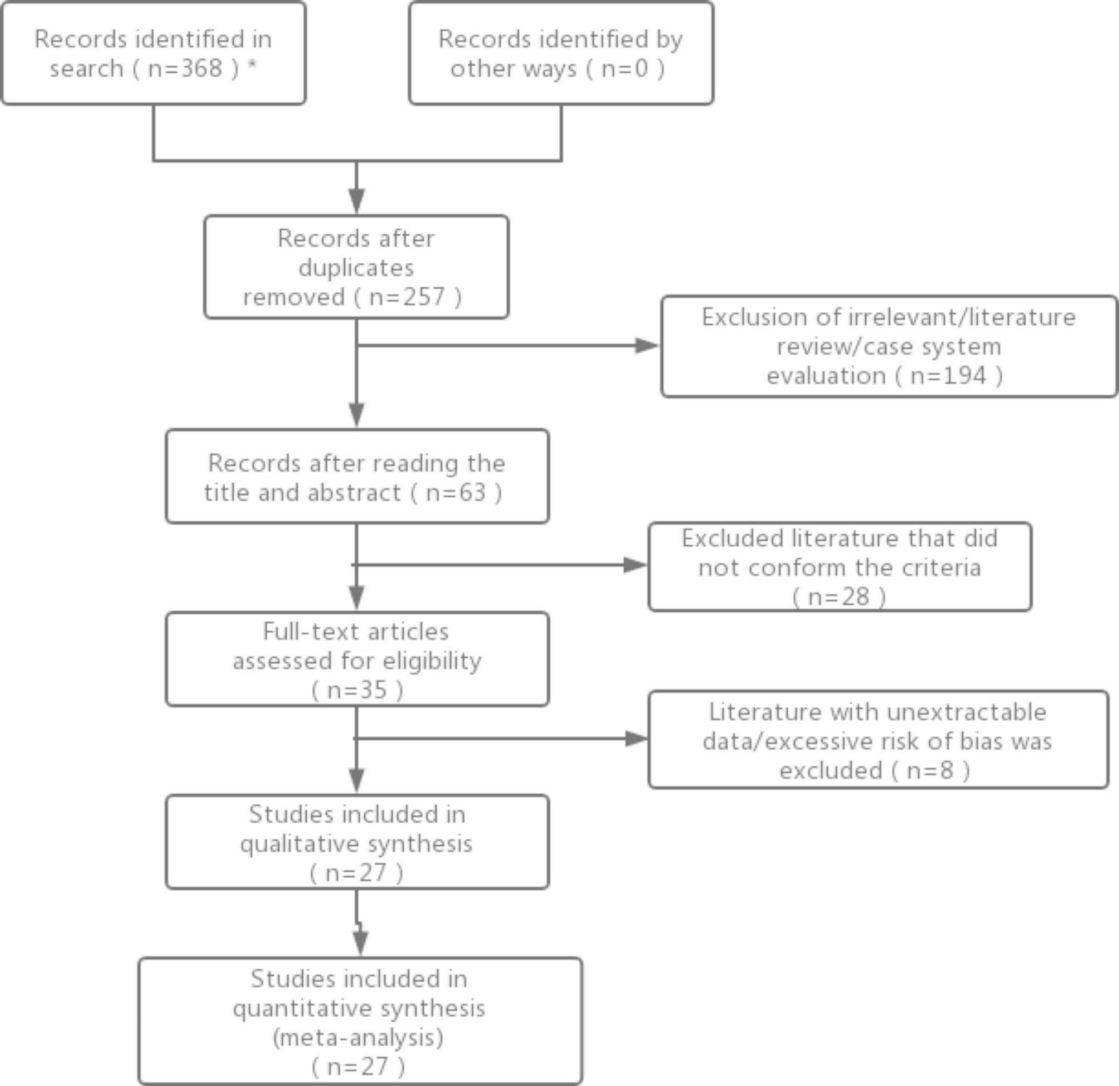
Literature screening process and results

According to the search results, a total of 368 studies were included in the initial stage, of which 111 duplicate literatures were deleted, and 194 studies of irrelevant, individual case, systematic overview, etc. were excluded from 257 studies screened after title and abstract reading. The full text of the remaining 63 studies was read, and 27 studies that met the inclusion criteria were finally identified [15–41] according to the inclusion/exclusion criteria, including 2891 patients, of which ^18^ F-PSMA-1007PET/CT involved 8 papers, ^18^ F-FDG PET/CT involved 9 papers, and ^68^Ga-PSMA PET/CT involved 11 papers. The general characteristics of the included studies were shown in Table 1. The specific literature screening process and results were shown in Fig. 2.

**Table 1:** General characteristics of the included literatures

First Author	Year	Country	Study type	Sample	Imaging agent	TP	FP	FN	TN
Kai, X.Z [15]	2020	China	Retrospective	21	^18^ F-PSMA-1007 PET/CT	15	2	1	3
Yu, L [16]	2018	China	Retrospective	104	^68^Ga-PSMA PET/CT	65	3	4	32
Miao, W [17]	2020	China	Prospective	71	^18^ F-FDG PET/CT	21	11	13	26
		China	Prospective	71	^18^ F-PSMA-1007 PET/CT	29	7	5	30
Yan, M.L [18]	2022	China	Prospective	46	^18^ F-PSMA-1007 PET/CT	35	2	4	5
Cui, P.J [19]	2018	China	Retrospective	33	^68^Ga-PSMA PET/CT	19	4	1	9
Liu, C [20]	2020	China	Retrospective	31	^68^Ga-PSMA PET/CT	14	4	1	12
Jiao, J [21]	2021	China	Retrospective + prospective	193	^68^Ga-PSMA PET/CT	86	13	8	86
Watanabe, H [22]	2010	Japan	Retrospective	43	^18^ F-FDG PET/CT	18	8	2	18
Xie Y [23]	2021	China	Retrospective	45	^68^Ga-PSMA PET/CT	28	2	4	11
Emmett, L [24]	2021	Australia	Prospective	291	^68^Ga-PSMA PET/CT	146	65	16	64
Tragardh, E [25]	2021	Sweden	Retrospective	39	^18^ F-PSMA-1007 PET/CT	37	2	0	0
Li, Y [26]	2021	China	Retrospective	46	^68^Ga-PSMA PET/CT	41	0	0	5
Morton, A [27]	2020	Australia	Retrospective	58	^68^Ga-PSMA PET/CT	51	0	2	5
Donato, P [28]	2019	Australia	Retrospective	144	^68^Ga-PSMA PET/CT	119	0	3	22
Pan, Y. C. H [29]	2018	Australia	Retrospective	239	^68^Ga-PSMA PET/CT	189	2	32	14
Hoffmann, MA [30]	2018	Germany	Prospective	25	^68^Ga-PSMA PET/CT	21	2	0	2
Pei, W [31]	2020	China	Retrospective	43	^18^ F-FDG PET/CT	31	5	4	3
Fu, M.Z [32]	2017	China	Retrospective	41	^18^ F-FDG PET/CT	31	3	4	3
Jiao, T [33]	2021	China	Retrospective	60	^18^ F-FDG PET/CT	29	5	4	22
Rousseau [34]	2019	Canada	Prospective	200	^18^ F-PSMA-1007 PET/CT	96	11	4	89
Song [35]	2020	USA	Prospective	200	^18^ F-PSMA-1007 PET/CT	90	15	10	85
Rowe [36]	2020	UK	Prospective	200	^18^ F-PSMA-1007 PET/CT	89	12	11	88
Wondergem [37]	2017	Netherlands	Retrospective	194	^18^ F-PSMA-1007 PET/CT	92	5	8	89
Damle [38]	2013	India	Retrospective	49	^18^ F-FDG PET/CT	23	0	9	17
Shiiba, M [39]	2012	Japan	Prospective	184	^18^ F-FDG PET/CT	58	18	36	72
Hwang, I [40]	2013	Korea	Retrospective	120	^18^ F-FDG PET/CT	20	65	3	32
Yang, Z [41]	2014	China	Retrospective	100	^18^ F-FDG PET/CT	13	25	7	55

*Literatures searched in each database: Pubmed (n = 13), Cochrane library (n = 18), Embase (n = 176), CNKI (n = 34), Wanfang (n = 80), VIP (n = 47).

### Quality assessment results

**Fig. 3:**
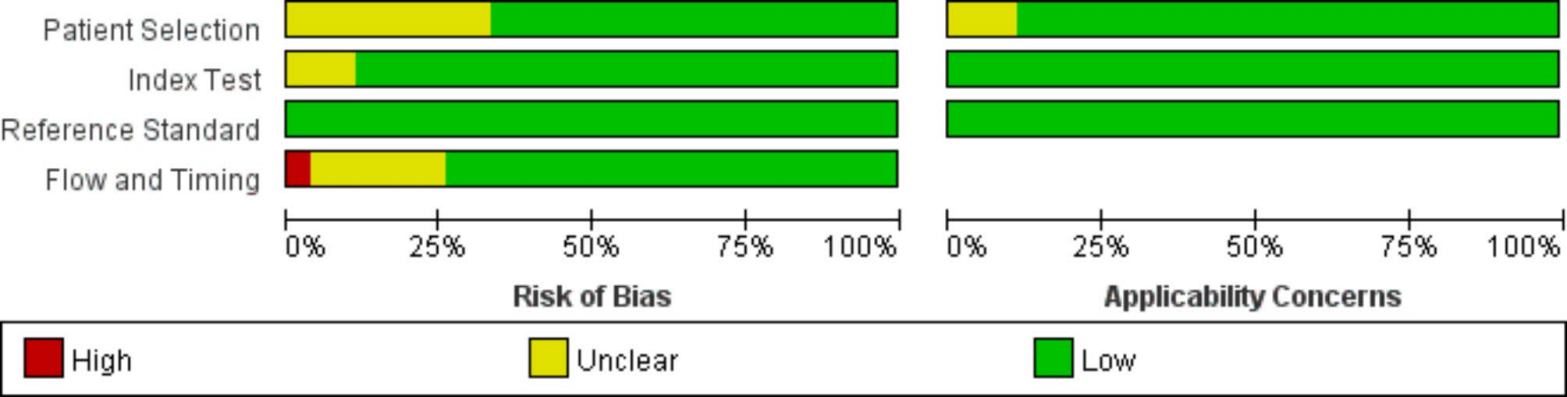
Quality assessment results of included literatures

The pathological biopsy was used as the only gold standard, and the quality assessment results of the QUADAS-2 scale showed in four areas, “unclear risk” was mainly observed in the first signal “Is there an appropriate time interval between the trial to be evaluated and the gold standard?“ in the “case flow and the time interval between the diagnostic tests and the implementation of the gold standards”. In addition, although a few literatures showed “high risk”, the overall quality of the included literatures was more credible, and the overall applicability was satisfactory, as shown in Fig. 3.

### Meta-analysis results

**Fig. 4:**
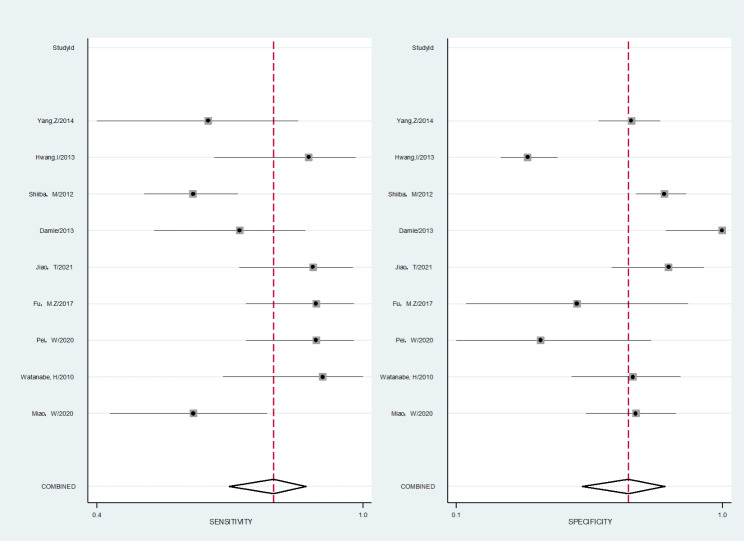
Forest plot of ^18^ F-PSMA-1007 PET/CT in the diagnostic efficacy of PCa

**Fig. 5:**
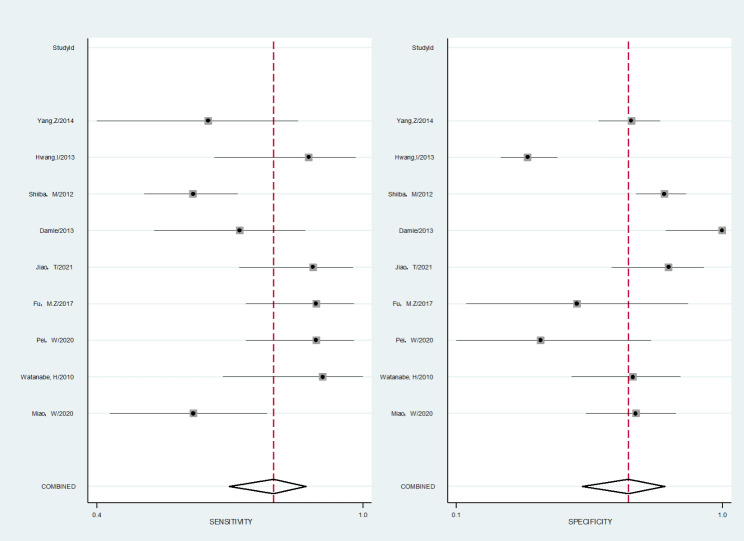
Forest plot of ^18^ F-FDG PET/CT in the diagnostic efficacy of PCa

**Fig. 6:**
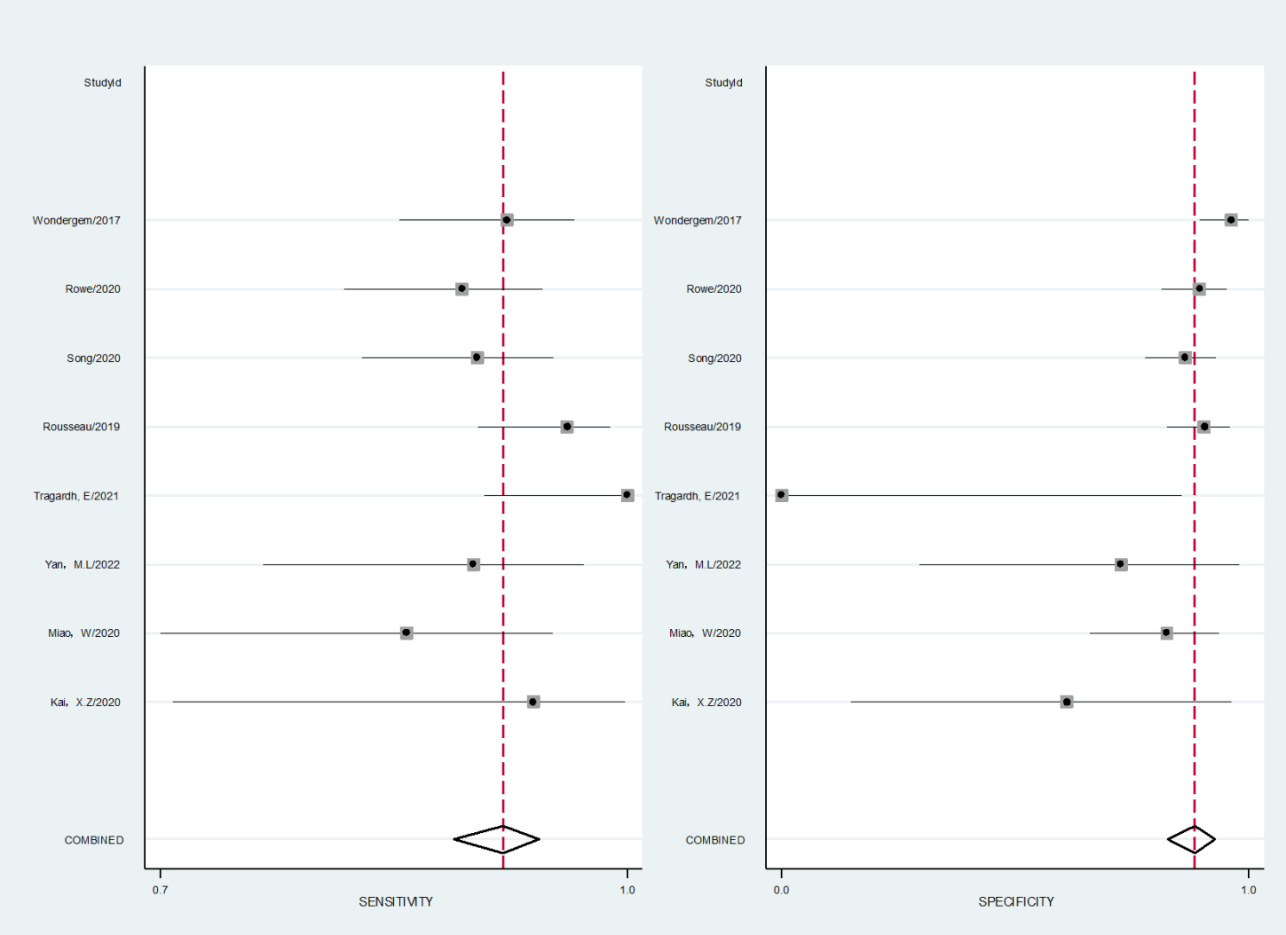
Forest plot of ^68^ F-Ga-PSMA PET/CT in the diagnostic efficacy of PCa

**Fig. 7:**
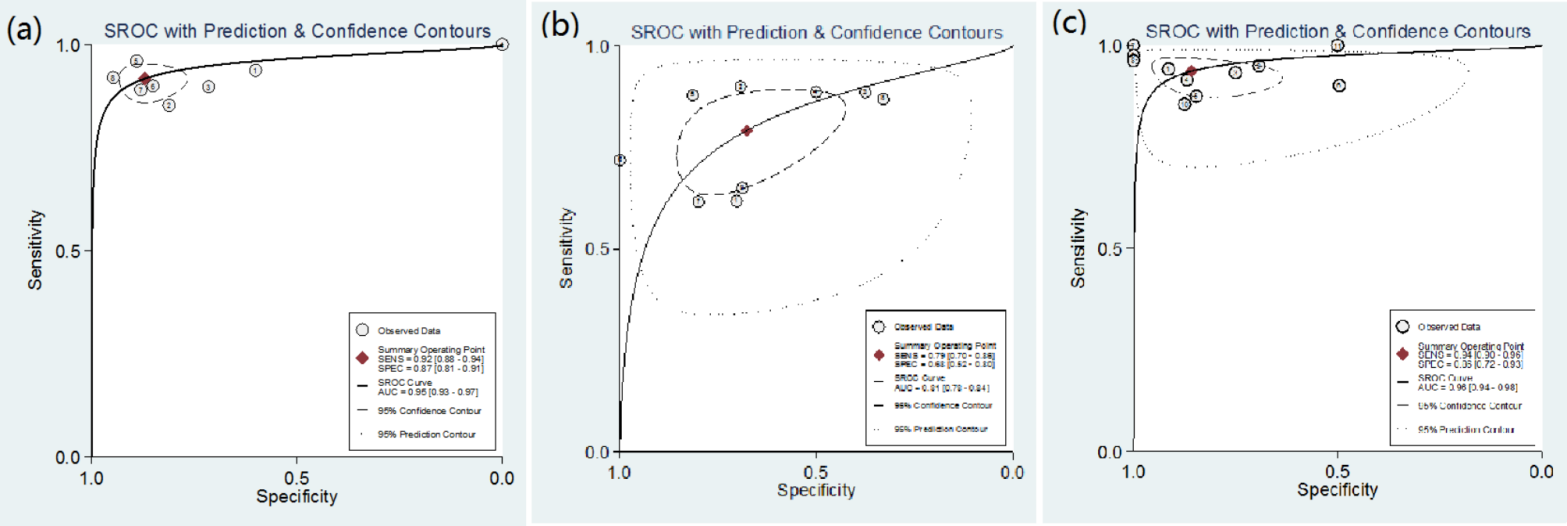
SROCs of ^18^ F-PSMA-1007 PET/CT (left), ^18^ F-FDG PET/CT (middle), and ^68^Ga-PSMA PET/CT (right)

The 27 literatures included in the study were pooled and analyzed, and the forest plots (Fig. 4, Fig. 5, Fig. 6) and SROCs (Fig. 7) were drawn for the three diagnostic methods of ^18^ F-PSMA-1007 PET/CT, ^18^ F-FDG PET/CT, and ^68^Ga-PSMA PET/CT. The results showed that the pooled sensitivities of ^18^ F-PSMA-1007 PET/CT, ^18^ F-FDG PET/CT, and ^68^Ga-PSMA PET/CT were 0.912 (95%CI: 0.883–0.936), 0.748 (95%CI: 0.698–0.795), and 0.916 (95%CI: 0.896–0.934), respectively; the pooled specificities were 0.878 (0.844–0.907), 0.639 (95%CI: 0.589–0.687), and 0.734 (95%CI: 0.685–0.779), respectively; the positive likelihood ratios were 6.335 (95%CI: 4.288–9.357), 2.282 (95%CI: 1.497–3.477), and 3.593 (95%CI: 2.986–4.323), respectively; the negative likelihood ratios were 0.878 (95%CI: 0.844–0.907), 0.374 (95%CI: 0.280–0.499), and 0.110 (95%CI: 0.083–0.144), respectively; the diagnostic odds ratios were 65.125 (95%CI: 34.059–124.53), 7.094 (95%CI: 4.091–12.301), and 29.722 (95%CI: 20.141–43.863), respectively; the area under the SPOC curve was 0.95 (95%CI: 0.93–0.97), 0.81 (95%CI: 0.78–0.84), and 0.96 (95%CI: 0.92–0.98), respectively.

### Heterogeneity analysis

Since Q = 0.068 (P = 0.483) and I^2^ = 0% in the ^18^ F-PSMA-1007 PET/CT heterogeneity test, Q = 35.148 (P = 0.000) and I^2^ = 94% in the ^18^ F-FDG PET/CT heterogeneity test, and Q = 11.472 (P = 0.002) and I^2^ = 83% in the ^18^Ga-PSMA PET/CT heterogeneity test, the random effects model was used. The Spearman correlation coefficient was used to explore the threshold effect, and the results showed that the Spearman correlation coefficients of ^18^ F-PSMA-1007 PET/CT (left), ^18^ F-FDG PET/CT (middle), and ^68^Ga-PSMA PET/CT were − 0.214 (P = 0.645), 0.377 (P = 0.318), and − 0.333 (P = 0.318), respectively, suggesting that there was no significant threshold effect.

### Meta regression analysis and subgroup analysis

**Fig. 8:**
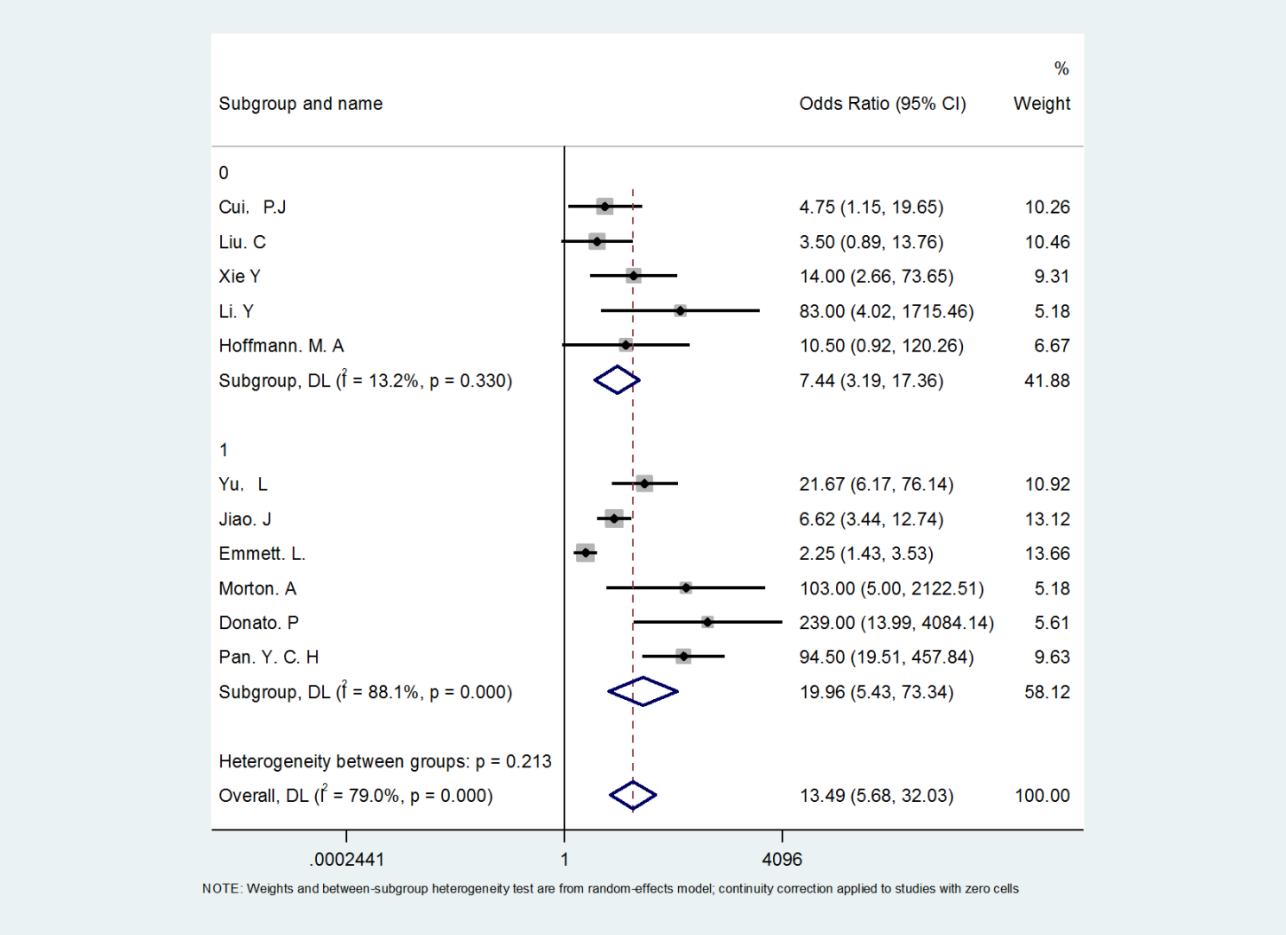
Subgroup analysis of the relevance to sample size in ^18^Ga-PSMA PET/CT study

In order to explore the potential sources of heterogeneity in this study, ^18^Ga-PSMA PET/CT (included literature n = 11 > 10) was subjected to the Meta regression analysis with the “Publication Year”, “Study Type”, “Sample Size” and “Publication Region/Country” as covariates. Since less than 10 papers related to ^18^ F-PSMA-1007 PET/CT and ^18^ F-FDG PET/CT were included in the study, the Meta regression analysis was not performed. The results of Meta regression analysis showed “Publication Year " (P = 0.911), “Study Type” (P = 0.556), “Sample Size” (P = 0.136), “Publication Region/Country” (P = 0.652), the P value of “sample size” is closer to 0.05, suggesting that the sample size may be the potential source of heterogeneity in ^18^Ga-PSMA PET/CT study, but the current evidence is not clear (P > 0.05). Therefore, a subgroup analysis of “Sample Size” was further conducted (0: n < 50, 1: n ≥ 50), and the results showed that the heterogeneity was related to the sample size (I^2^ = 79%, P = 0.000) (Fig. 8).

### Clinical analysis

**Fig. 9:**
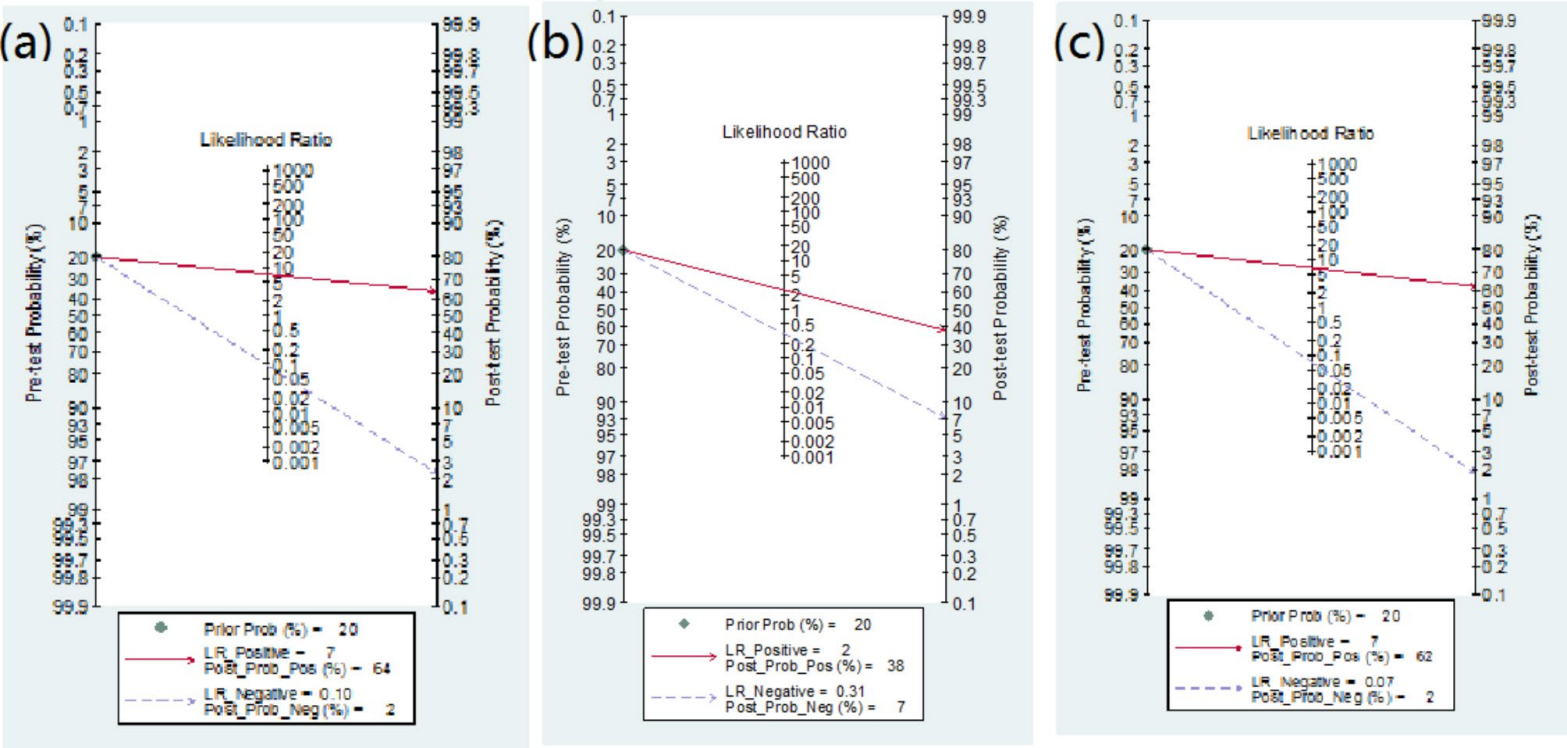
Fagan plot of ^18^ F-PSMA-1007 PET/CT **(a)**, ^18^ F-FDG PET/CT **(b)** and ^18^Ga-PSMA PET/CT **(c)**

Post-test probability (the estimated incidence after the diagnostic test) was analyzed using Fagan plots. The results showed that when the pre-test probability of diagnosing PCa was defined as 0.20, the PPPs of ^18^ F-PSMA-1007 PET/CT, ^18^ F-FDG PET/CT, and ^68^Ga-PSMA PET/CT were 64%, 38%, and 62%, respectively (Fig. 9).

### Publication bias test

**Fig. 10:**
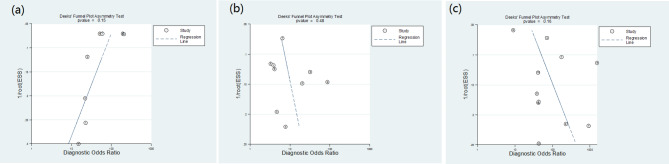
Funnel plot of ^18^ F-PSMA-1007 PET/CT **(a)**, ^18^ F-FDG PET/CT **(b)** and ^18^Ga-PSMA PET/CT **(c)**

The results of Deek’s funnel plot test showed that the related studies of ^18^ F-PSMA-1007 PET/CT, ^18^ F-FDG PET/CT, and ^68^Ga-PSMA PET/CT were almost symmetrical, and the P values were 0.160, 0.482, and 0.153, respectively, indicating that there was no significant in publication bias, as shown in Fig. 10.

## Discussions

In this study, a meta-analysis was carried out for the diagnostic efficacy of PET/CT with different imaging agents, and the results suggested that ^18^ F-PSMA-1007 PET/CT and ^68^Ga-PSMA PET/CT had higher diagnostic efficacy of prostate cancer compared with ^18^ F-FDG PET/CT, among which ^68^Ga-PSMA PET/CT was slightly higher in the sensitivity of the diagnosis of prostate cancer, while ^18^ F-PSMA-1007 PET/CT may have higher efficacy in specificity and confirmed positive rate.

Meta-analysis results showed that the SENs of ^18^ F-PSMA-1007 PET/CT, ^18^ F-FDG PET/CT, and ^68^Ga-PSMA PET/CT were 0.912, 0.748, and 0.916, respectively, and the SPEs were 0.878, 0.639, and 0.734, respectively, suggesting that ^18^ F-PSMA-1007 PET/CT and ^68^Ga-PSMA PET/CT were superior to ^18^ F-FDG PET/CT in the diagnostic accuracy, and ^68^Ga-PSMA PET/CT showed higher sensitivity in the diagnosis of PCa. While ^18^ F-PSMA-1007 PET/CT showed higher specificity. Zhou et al. [42] also concluded that ^18^ F-FDG PET/CT has lower accuracy than other methods in the comparison of the diagnostic efficacy of PET/CT with different imaging agents. In addition, the DORs of the other three methods were 65.125, 7.094, and 29.722, respectively, suggesting that ^18^ F-PSMA-1007 PET/CT had higher differentiation. The LR + values were 6.335, 2.282, and 3.593, respectively, and the LR- values were 0.878, 0.374, and 0.110, respectively, indicating that ^18^ F-PSMA-1007 PET/CT had higher PCa positive diagnostic value, but ^68^Ga-PSMA PET/CT had higher accuracy in the negative monitoring results. The areas under the SPOC curves were 0.95, 0.81, and 0.96, respectively, indicating that ^18^ F-PSMA-1007 PET/CT and ^68^Ga-PSMA PET/CT had higher diagnostic efficacy. Analysis of Fagan plots showed that when the pre-test probability of diagnosing PCa was defined as 0.20, the PPPs were 64%, 38%, and 62%, respectively, i.e., when the probability of PCa was 20% based on clinical manifestations, the PCa diagnosis probability of the three PET/CT methods were 64%, 38%, and 62%, respectively, suggesting that ^18^ F-PSMA-1007 PET/CT may detect other PCa-related lesions, which was consistent with the findings of Kuten et al. [43].

The heterogeneity analysis in this study found that the “Sample Size” may be a potential source of bias in the meta-analysis of ^68^Ga-PSMA PET/CT. Since the heterogeneity test found that there was significant heterogeneity in the results of the three groups, the ^68^Ga-PSMA PET/CT that met the requirements of Meta regression analysis was analyzed. Although the results did not show the potential source of heterogeneity at P < 0.05, the P value of “Sample Size” was relatively small, so this factor was highly suspected as a potential source of heterogeneity. However, this meta-regression analysis did not yield satisfactory results due to the effects of the number of included literatures (just meeting the requirement of Meta regression literatures ≥ 10) and the quality of the literatures. Therefore, a subgroup analysis of “Sample Size” was further conducted (0: n < 50, 1: n ≥ 50), and the results validated that the heterogeneity was related to the sample size (I^2^ = 79%, P = 0.000). Therefore, the heterogeneity analysis in this study was more reliable.

This study has certain limitations: (1)The included literatures lack multi-center large-sample studies, which has a certain impact on the quality of the literatures and the source of heterogeneity, and may affect the accuracy of the results; (2)The time interval between imaging examination and gold standard examination was not clear in many included literatures, so various biases cannot be avoided; (3) Since there were unclear time intervals between the imaging test and the gold standard in many included literatures, many biases cannot be avoided; (4) The included studies have certain clinical heterogeneity, such as inconsistency in PET/CT models and operators, which may become sources of heterogeneity; (5) Subtypes of prostate cancer and differences in diagnostic efficacy of different imaging agents were not mentioned in the included literature. Therefore, the impact of PCa subtypes was not investigated in this study.

## Conclusion

In conclusion, ^18^ F-PSMA-1007 PET/CT and ^68^Ga-PSMA PET/CT had higher diagnostic efficacy of PCa compared with ^18^ F-FDG PET/CT, among which ^68^Ga-PSMA PET/CT was slightly higher in the sensitivity of the diagnosis of PCa, while ^18^ F-PSMA-1007 PET/CT may have higher efficacy in specificity and confirmed positive rate. However, due to the limitations of the quality of the included samples and literatures, the above conclusions still should be further validated by expanding the sample size and improving the quality.

## Data Availability

Not applicable.

## Abbreviations

QUADAS-2: Quality Assessment of Diagnostic Accuracy Studies
PCa: Prostate cancer
PSA: Serum prostate-specific antigen
PSMA: Prostate-specific membrane antigen
TP: True positive value
FP: False positive value
TN: The true negative value
FN: False negative value
SEN: Pooled sensitivity
SPE: Pooled specificity
LR +: Positive likelihood ratio
LR-: Negative likelihood ratio
DOR: Diagnostic odds ratio
PPP: Positive posterior probability
SROC: Summary Receiver Operating Characteristic
